# Dad's Diet Shapes the Future: How Paternal Nutrition Impacts Placental Development and Childhood Metabolic Health

**DOI:** 10.1002/mnfr.70261

**Published:** 2025-09-06

**Authors:** David A. Skerrett‐Byrne, Anne‐Sophie Pepin, Katharina Laurent, Johannes Beckers, Robert Schneider, Martin Hrabě de Angelis, Raffaele Teperino

**Affiliations:** ^1^ Institute of Experimental Genetics Helmholtz Zentrum München German Research Center for Environmental Health Neuherberg Germany; ^2^ German Center for Diabetes Research (DZD) Neuherberg Germany; ^3^ Infertility and Reproduction Research Program Hunter Medical Research Institute New Lambton Heights NSW Australia; ^4^ School of Biomedical Sciences and Pharmacy College of Health Medicine and Wellbeing The University of Newcastle Callaghan Awabakal Country NSW Australia; ^5^ Institute of Functional Epigenetics Helmholtz Zentrum München Neuherberg Germany; ^6^ Chair of Experimental Genetics TUM School of Life Sciences Technische Universität München Freising Germany; ^7^ Faculty of Biology Ludwig‐Maximilians‐Universität München Planegg‐Martinsried Germany; ^8^ Institute of Experimental Genetics German Mouse Clinic Helmholtz Zentrum München German Research Center for Environmental Health (GmbH) Neuherberg Germany

**Keywords:** epigenetics, inheritance, nutrient, paternal preconception health, placenta, ShinyApp

## Abstract

Early‐life programming is a major determinant of lifelong metabolic health, yet current preventive strategies focus almost exclusively on maternal factors. Emerging experimental and preclinical data reveal that a father's diet before conception, particularly high‐fat intake, also shapes offspring physiology. Here, we synthesize the latest evidence on how such diets remodel the sperm epigenome during two discrete windows of vulnerability: (i) testicular spermatogenesis, via DNA methylation and histone modifications, and (ii) post‐testicular epididymal maturation, where small non‐coding RNAs are selectively gained. We examine how these epigenetic signals influence pregnancy, placental development, and ultimately, metabolic trajectories in progeny. To extend published work, we sourced publicly available diet‐induced sperm epigenome datasets and provide new potential connections of these changes to genes governing placental development, vascularization and size using the International Mouse Phenotyping Consortium data. Moreover, we further interrogate these overlaps with intricate in‐silico analyses to examine their potential consequences. To foster meaningful interactions with these findings, we have developed a web application for ease (ShinySpermPlacenta). Collectively, these findings support a biparental model of preconception care and position the sperm epigenome as a promising tractable biomarker platform for personalized paternal nutrition counselling aimed at improving fertility and reducing intergenerational metabolic disease risk.

## Graphical abstract

This review highlights the emerging connection between paternal nutrition‐induced sperm epigenetic changes and placental phenotypes that may influence offspring metabolic health. An accompanying interactive ShinyApp allows exploration of these sperm–placenta gene networks, a framework for future research seeking to explore candidate biomarkers or intervention targets for paternal dietary health.

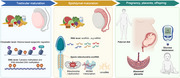

## 1 Introduction

The early life environment plays a critical role in shaping an individual's long‐term health trajectory, influencing susceptibility to metabolic disorders, obesity, and diabetes [1, 2]. Increasing evidence is pointing to the importance of a father's health prior to conception, which can exert profound effects on pregnancy, offspring development, and disease risk.[1, 3] Although maternal health and nutrition have long been recognized as key determinants of fetal growth and metabolic programming [4, 5], the contribution of paternal factors has been historically underappreciated. However, accumulating evidence now highlights paternal preconception health as a significant, yet often overlooked, determinant of offspring metabolic and developmental outcomes [6].

One of the most striking examples of developmental programming is the intergenerational transmission of metabolic disease risk. Children born to obese or diabetic parents face a significantly higher likelihood of developing obesity, insulin resistance, and cardiometabolic disorders later in life [7, 8]. This relationship is well‐documented in maternal‐fetal health, but recent studies have demonstrated that paternal obesity and poor dietary habits can similarly predispose offspring to metabolic dysfunction [9, 10]. In the European context, we are now at a stage where over 50% of adults are classified as overweight or obese [11]; therefore, understanding the mechanisms underlying intergenerational health risks is of critical importance. Moreover, the economic burden of obesity‐related diseases, including the rising cases of type 2 diabetes (T2DM) and metabolic syndromes, is estimated to cost the European healthcare systems billions annually [12], underscoring the urgent need for preventative strategies that extend beyond maternal health to include paternal factors as well.

Despite the mounting experimental and preclinical evidence supporting a role for paternal preconception health, scientific and public health discussions remain largely maternal‐centric, primarily focused on maternal nutrition, lifestyle, and health interventions, while paternal contributions to offspring health remain relatively understudied [13, 14]. This knowledge gap presents an opportunity to rebalance the narrative around paternal contributions and recognize sperm as not merely a vehicle for genetic information but as an epigenetic vector capable of transmitting environmental and metabolic cues to the next generation. A growing body of work now suggests that paternal dietary habits can reprogram sperm at the molecular level, leading to potentially heritable epigenetic modifications [10]. Environmentally‐induced changes in sperm DNA methylation [15, 16], histone modifications [17, 18], and small non‐coding RNAs (sncRNAs) [19, 20] can influence embryonic gene expression, placental development, and nutrient transfer, ultimately shaping the metabolic future of the offspring [21, 22, 23]. Although these effects were initially thought to be permanently set during spermatogenesis (testicular contributions), new experimental and preclinical evidence highlights epididymal maturation (post‐testicular) as a second critical window where sperm can acquire epigenetic information, driven by environmental and dietary stressors [19, 20, 24, 25].

This review will explore the emerging role of sperm epigenetics as a key mediator of paternal dietary effects on offspring metabolic health. Although important, this review will not focus on non‐germ cell factors such as seminal fluid (reviewed here, [26, 27, 28]) and paternal behavioral and social influences on shaping offspring outcomes, often through indirect maternal mediation (reviewed here, [29, 30]). Specifically, we will discuss how paternal nutrition can reprogram the sperm epigenome, and the downstream consequences for embryonic development, placental function, and offspring metabolic programming. Moreover, through *in‐silico* analyses we will draw new connections between published diet‐induced sperm epigenetic changes to important genes known to regulate placental phenotypes. By synthesizing recent findings, and deploying an interactive ShinyApp, we aim to highlight the importance of paternal preconception health in shaping the developmental and placental origins of metabolic disease and advocate for greater inclusion of paternal factors in reproductive and public health strategies.

## 2 Methods

### 2.1 HFD Sperm Epigenetic Data Sourcing and miRNA Target Gene Prediction

**FIGURE 1:**
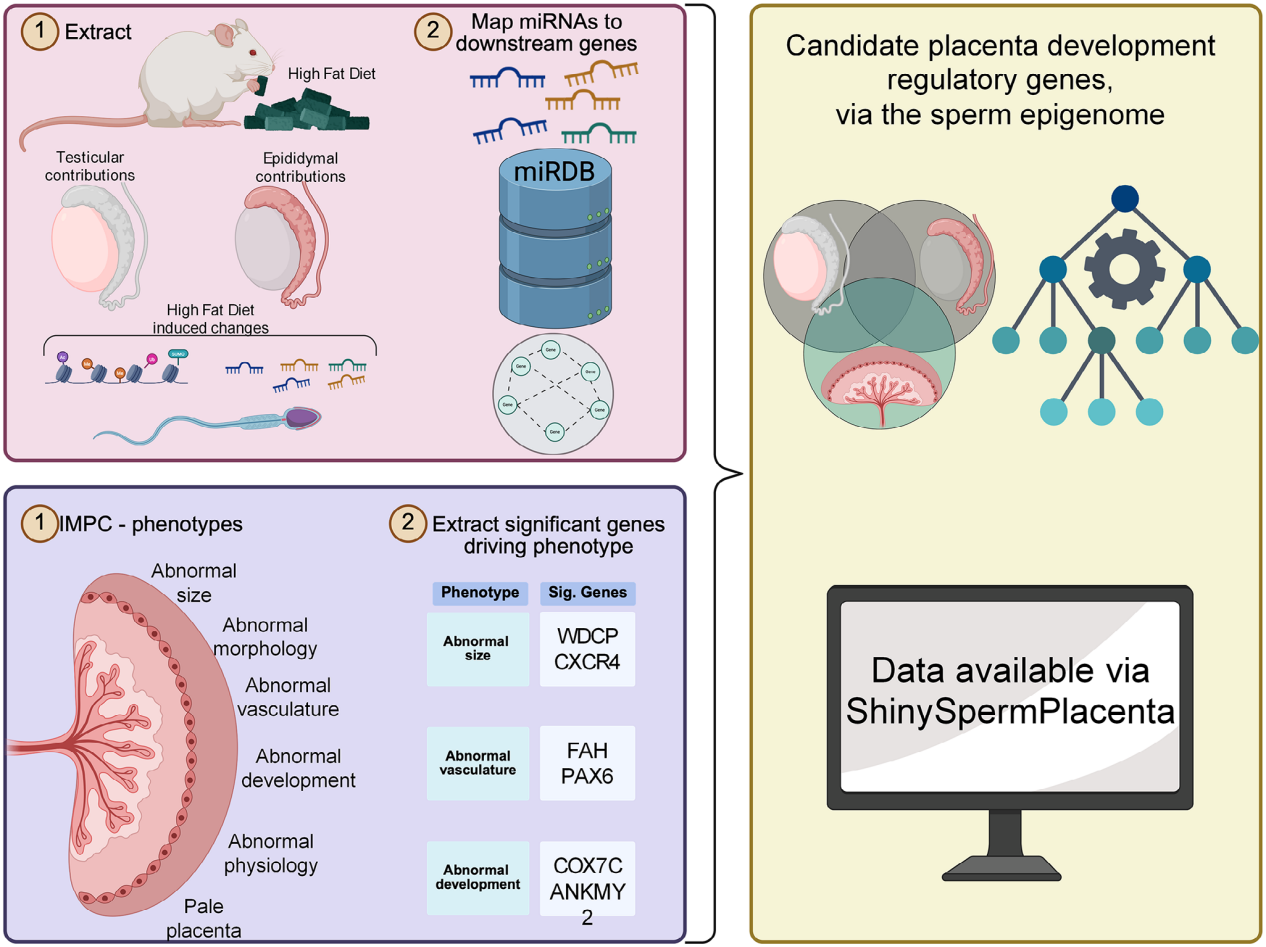
Linking paternal diet to placental development workflow. (A) Mouse studies that implemented high‐fat diets where sourced and significant changes to sperm chromatin sncRNAs were extracted. The microRNA target prediction database (miRDB) was used to identify all downstream target genes. (B) In parallel, the International Mouse Phenotyping Consortium (IMPC) was mined for placenta‐related phenotypes, and all significant gene drivers were extracted. (C) Candidate regulators of placental development via the sperm epigenome were identified and subjected to in silico analyses with ingenuity pathway analysis (IPA). The final results are made accessible through an interactive ShinyApp, ShinySpermPlacenta (https://reproproteomics.shinyapps.io/ShinySpermPlacenta/).

To pinpoint epigenetic changes that arise in the testis versus those added later during epididymal maturation, we performed a structured PubMed search using keywords including “*sperm*,” “*high fat diet*,” “*transcriptomics*,” “*epigenetics*,” “*histone methylation*,” and “*epigenetic inheritance*” from 2010 to 2025. Studies were refined to those that implemented a high‐fat diet (HFD) regime and employed methods such as transcriptomic, sncRNA sequencing, and ChIP‐seq, with the data publicly available. This search strategy yielded two testis‐focused studies [17, 18], that is, exposure time coincides with at least a full round of spermatogenesis and epididymal transit, and five epididymal studies [19, 31, 32, 33, 34], that is, those in which exposure only coincides with epididymal transit (Figure 1).

From the two HFD testis studies, as previously described [17, 18], we selected the top 10% genomic regions contributing to the principal components associated with HFD that annotate to promoter regions, compiling 1844 genes showing histone methylation changes (Table S1). From the five HFD studies that interrogated mature epididymal sperm sncRNA cargo, we identified 148 microRNAs (miRNAs) whose abundance was significantly increased due to HFD (Log_2_FC ≥ 1, FDR ≤ 0.05) (Figure 1). Employing the miRNA target gene prediction database (miRDB) [24, 35], we mapped 90/148 miRNAs to 5780 high‐confidence downstream target genes (strict prediction score ≥80/100 [24]) (Table S2). Tables S1 and S2 list, for each gene or miRNA, the specific study (or studies) from which it was derived.

### 2.2 The International Mouse Phenotyping Consortium Phenotype Extraction

In parallel, we examined the catalogue of knockout mouse lines from the International Mouse Phenotyping Consortium (IMPC) [36, 37], a global initiative to systematically knock out every protein‐coding gene in the mouse genome and assess the resulting phenotypes through standardized physiological tests across a wide range of biological systems. This large‐scale effort is designed to elucidate gene function and its impact on organismal biology, with all resulting data made publicly available via the IMPC portal (https://www.mousephenotype.org/). Leveraging this vast resource, we extracted all genes significantly associated (*p* ≤ 0.0001) with placental phenotypes, both homozygous and heterozygous pups, including abnormalities in size, morphology, vasculature, physiology, coloration (e.g., pale appearance), and chorioallantoic fusion (Figure 1, Tables 1 and S3).

**TABLE 1:** The available placenta phenotypes and the number of significant genotype‐phenotype associations on the IMPC.

IMPC phenotype	Significant genes
Placenta morphology	140
Placenta vasculature	138
Placenta size	96
Chorioallantoic fusion	22
Placenta development	6
Pale placenta	2
Placenta color	2

### 2.3 Cross‐Dataset Integration and Ingenuity Pathway Analysis

To identify potential mechanistic links between sperm epigenomic contributors and placental development, we cross‐referenced the lists of significant genes identified from testicular and epididymal sources (Tables‐S1 and S2) with genes associated with placental phenotypes (Table S3). Overlaps were assessed using [38] (Venny 2.1).

The overlaps gene sets from testicular contributions (19 genes) and those derived from the epididymis (56 genes) were subject to analysis using Ingenuity Pathway Analysis software (IPA; Qiagen, Hilden, Germany) as previously described [24, 39, 40, 41, 42]. These gene sets were analyzed on the basis of predicted pathways and downstream molecular functions using the IPA *p* value enrichment score [43], with a stringency criterion of ‐log10 *p* value of ≥1.3 for each analysis. Additionally, genes were annotated to known subcellular localization and functional classification, excluding those without clear categorization (“*other*”).

### 2.4 ShinyApp Development

Following the design principles established in ShinySperm [44] and ShinySpermKingdom [45], we developed a Shiny web application, ShinySpermPlacenta, to provide a user‐friendly environment for exploring and interpreting the datasets presented in this review. The app was developed using the shiny package (v1.10.0) in R (v4.5.0, released 2025‐04‐11) via RStudio (v2025.05.0+496). Additional packages supporting the app's functionality and visual presentation include DT, eulerr, ggplot2, openxlsx, plotly, readxl, reshape2, RColorBrewer, and shinydashboard.

## 3 Paternal Nutrition Shaping Sperm Epigenetics

Sperm cells are the primary drivers of paternal influence on offspring development and are themselves vulnerable to environmental and nutritional cues. There are two critical windows during sperm development where their epigenome remains highly plastic: spermatogenesis [46] and epididymal maturation [47].

Spermatogenesis marks the first phase of sperm's transformation into a fertilization‐competent cell, occurring within the testes, where diploid spermatogonia progressively differentiate into haploid spermatozoa [48]. During this process, the male germ cells undergo extensive epigenetic reprogramming, including dynamic changes in DNA methylation, RNA content [49], and histone content and modifications [46]. These epigenetic marks, once considered to be fully erased and re‐established de novo, are now recognized to be sensitive to paternal diet and metabolic status [17]. Recent work demonstrated that paternal obesity, induced by an HFD, leads to widespread alterations in sperm histone methylation, particularly at histone H3 lysine 4 trimethylation (H3K4me3)​ [17]. These differentially enriched regions localized at the promoter of genes linked to metabolic regulation, inflammatory responses, and developmental pathways, and occurred at genes expressed in the pre‐implantation inner‐cell mass (ICM), trophectoderm (TE), as well as in the placenta [17, 18]. Notably, the offspring of obese sires exhibited metabolic dysfunction, suggesting that H3K4me3 may link paternal diet to intergenerational inheritance of metabolic disease via the placenta. Similarly, de Castro Barbosa et al. investigated the multigenerational impact of a paternal HFD on sperm DNA methylation and sncRNAs, and identified several differentially methylated regions associated with >90 genes involved in glucose metabolism and insulin signaling [50]. Although these DNA methylation changes were correlated with offspring gene expression profiles, a direct causal relationship was not demonstrated. The offspring (F1) from HFD‐fed fathers initially received a chow diet, but a subgroup was later re‐exposed to HFD during adulthood, exhibiting altered metabolic phenotypes, including reduced body weight and pancreatic beta cell mass. Intriguingly, sperm from chow‐fed F1 offspring displayed DNA methylation and sncRNA profiles similar to their HFD‐exposed fathers, suggesting persistent epigenetic memory. Crucially, chow‐fed F1 males retained epigenetic changes and transmitted metabolic vulnerability to the F2 generation, where these epigenetic and metabolic changes could be exacerbated upon subsequent dietary challenge. These studies and others emphasize that disruptions at this stage can have downstream effects on embryo development and offspring metabolic health, laying the groundwork for intergenerational or even transgenerational influences.

A defining feature of these morphologically specialized cells is the condensation of chromatin, achieved through the replacement of histones by protamines, which both protects sperm DNA from damage and renders the genome transcriptionally silent by eliminating the cell's capacity for gene transcription and protein synthesis. [51]. Despite their already profound structural specialization, sperm cells exiting the testes remain functionally immature, lacking progressive motility and the capacity to engage in productive interactions with the ovum [52]. These essential functional attributes are acquired progressively during transit through the epididymis, a highly specialized region of the male excurrent duct system [47]. Historically underappreciated, the epididymis plays a pivotal role in shaping the epigenetic cargo of maturing sperm cells. As sperm navigate this highly compartmentalized environment, they undergo substantial molecular remodeling, including alterations to their proteomic [39], lipid, and epigenetic landscapes [53]. Remarkably, sperm lose over 50% of their proteome during epididymal transit, acquiring only a selective repertoire of essential proteins [39]. This remodeling is orchestrated by the epididymal epithelium [54], which secretes a diverse array of signaling molecules, extracellular vesicles (EVs, epididymosomes), and metabolic factors into the epididymal lumen [47, 52]. These extrinsic cues, primarily concentrated in the proximal (caput) epididymis, not only facilitate sperm functional maturation but also contribute to their final epigenetic cargo through the exchange of diverse small non‐coding RNAs (sncRNAs; e.g., microRNAs [miRNAs] and piwi‐interacting RNAs [piRNAs]) [20, 24, 25, 53]. This stage of post‐testicular maturation provides an additional layer of epigenetic regulation that can be modulated by paternal nutrition.

Environmental or dietary factors impacting epididymal function can alter sperm sncRNA payload, with consequences to reprogramming embryonic and placental development [20, 25]. Indeed, Sharma et al. demonstrated that tRNA fragments (tRFs) in sperm are highly responsive to dietary shifts, with a low‐protein diet leading to increased levels of specific tRFs, notably tRF‐Gly‐GCC, in mature sperm. Injection of these small RNAs or synthetic tRF‐Gly‐GCC into zygotes can modulate gene expression in early embryos, particularly repressing MERVL‐associated transcripts [55]. In a complementary study, HFD in male mice induced alterations in sperm tRFs, identifying them as key mediators of metabolic inheritance, influencing glucose homeostasis and lipid metabolism in offspring [56]. Further, patients subjected to a week‐long healthy diet regimen followed by a week of high sugar intake, induced a rapid increase in tRNA‐derived small RNA (tsRNA) in their sperm and increased sperm motility [57]. Similarly, work in *Drosophila* demonstrated that just 2 days of paternal sugar feeding altered the sperm epigenome and induced metabolic reprogramming in offspring [58]. These findings underscore the capacity for paternal dietary influences to rapidly reshape the sperm epigenome for example via sncRNAs, with consequences that extend beyond fertilization to metabolic reprogramming. Extending this concept to environmental dietary contaminants, Trigg et al. investigated acrylamide exposure in mice, a dietary toxin commonly found in processed and fried foods, pinpointing the caput epididymis epithelium as the key site of sperm RNA remodeling [20]. Using a one week exposure model, this study demonstrated the epithelium proteome responds to acrylamide through upregulation of several transcription factors (e.g., NR3C1, glucocorticoid receptor) [24, 59], driving the biogenesis of these stress‐responsive sncRNA, which are subsequently packaged in epididymal derived extracellular vesicles (epididymosomes) [60, 61], serving as the conduit for sncRNA exchange of somatic‐germline communication. In a complementary heat stress mouse model [25], the impact of a one‐week whole‐body heatwave simulation was examined on the sperm epigenome. Their findings highlighted that sperm sncRNAs, particularly miRNAs, were significantly dysregulated following heat exposure, again reinforcing the role of the epididymis as a key site of epigenetic remodeling. This heat‐induced shift in sperm miRNA content correlated with profound post‐fertilization consequences, leading to accelerated embryonic development, aberrant blastocyst hatching, and significant changes in placental morphology. Most strikingly, the labyrinth zone of the placenta, the critical interface for maternal‐fetal nutrient exchange, was significantly altered, suggesting that paternal heat stress influences not just embryo development but also placental efficiency and offspring growth trajectories.

Beyond carbohydrate excess, other nutrient classes have also been shown to remodel the human sperm sncRNA landscape. A recent short‑term supplementation trial in healthy volunteers demonstrated that a 6‐week dietary intervention of extra virgin olive oil (65% fatty acids as oleic acid) and a daily fruit drink (containing marine omega‐3 acid and vitamin D), altered the abundance of >120 sncRNAs in human sperm, specifically tRFs, miRNAs, and piRNAs, targeting genes important in fatty acid metabolism [62]. Likewise, a comparison of lean versus obese men revealed 47 significantly differentially expressed miRNAs together with widespread shifts in tRF and piRNA abundance (collectively 326), and notably, histone retention profiles were unaltered between these groups [63]. Collectively, these emerging human data indicate that diverse macro‑ and micronutrients, as well as overall adiposity, are sensed along the male reproductive tract and relayed into a plastic sncRNA signature that may impart specific dietary information to the embryo.

Lastly, an emerging interest in how the epididymis is shaping the sperm RNA cargo is through sperm‐borne mitochondrial RNAs. Despite sperm cells being transcriptionally and translationally quiescent, new evidence indicates that during epididymal transit, sperm mitochondria can still transcribe and release fragments of mitochondrial RNA (mt‐tRNA) [19]. Utilizing a HFD mouse model and clinical data, Tomar et al. [19] eloquently illustrates how paternal diet can dysregulate sperm mitochondrial function, driving the expression of diet‐induced sperm‐borne mt‐tRNAs, which are transferred to the oocyte at fertilization, influencing not only early embryo gene expression but also leading to a robust glucose intolerance phenotype in their male offspring. Notably, the authors followed up their preclinical work in young healthy adults (19–21 years old, BMI 19.7–31.7) and found that mt‐tsRNAs were the only sperm sncRNA biotype whose abundance rose in a linear, positive association with BMI.

Taken together, these two windows of susceptibility demonstrate the remarkable plasticity of the sperm epigenome and offer opportunities where paternal nutrition can reshape it, ultimately influencing embryonic development, placental function (explored in Sections 4 and 5), and subsequently setting the trajectory of offspring metabolic health. Understanding how these processes intersect will provide the field with a framework for identifying critical paternal factors with implications for human reproductive health and metabolic disease prevention.

## 4 Placental Development and Early Nutrient Programming

**FIGURE 2:**
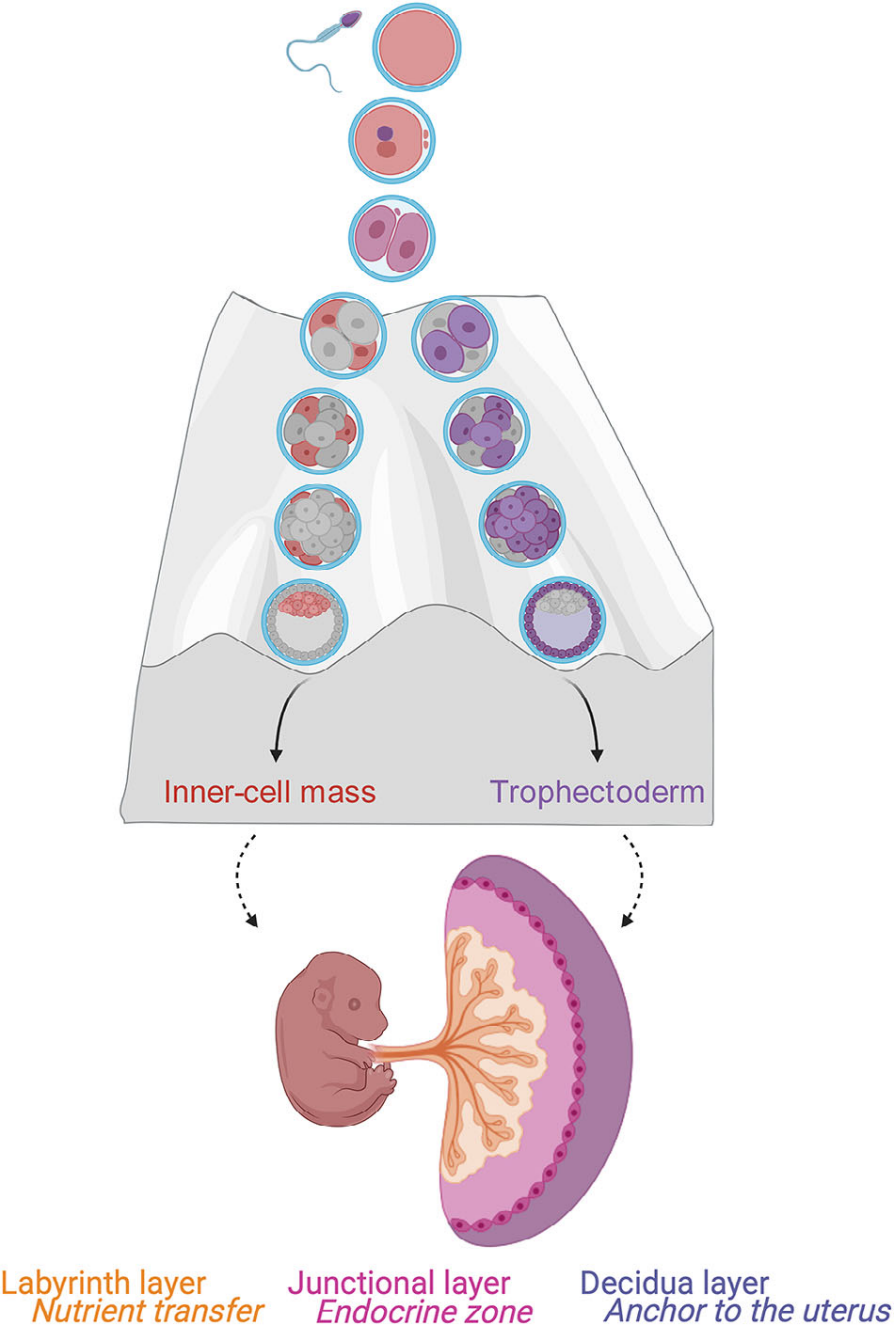
Early developmental potential biases and placental zonation. Schematic representation of early mouse embryonic development from fertilization to placental formation. Upon fusion of the sperm and oocyte, the embryo progresses through the 2‐cell, 4‐cell, 8‐cell, and morula stages to the blastocyst. Cell developmental potential biases towards the inner‐cell mass (ICM) or the trophectoderm (TE) are marked in red and in purple, respectively. The mature placenta is depicted with its three functional layers (labyrinth zone (orange) for nutrient exchange, junctional zone (pink) for endocrine activity, and decidua (purple) for structural support.

The placenta is a transient organ that supports embryonic development and fetal growth throughout gestation. In mice, upon fertilization, the embryo undergoes several rounds of cellular division and differentiation that introduce cell fate priming through regulatory elements and lineage‐specifying transcription factors, providing developmental potential towards either the ICM or the TE lineages (Figure 2) [64]. The first definitive lineage specification occurs at the early blastocyst stage where the ICM and the TE form [65]. The ICM will eventually divide into the epiblast and the primitive endoderm, precursors of the embryo and yolk sac, respectively. The implantation of the blastocyst then triggers a cascade of molecular, endocrine, and immune‐related processes that permit endometrial decidualization, where stromal cells acquire specialized secretory capacities [66, 67]. Impairment of this critical process have been linked to placental failure, infertility, and obstetrical complications. The first placental cell segregation occurs where the TE further separates into the extra‐embryonic ectoderm and the ectoplacental cone. Upon gastrulation, the amnion, chorion, and allantois form extra‐embryonic mesoderm originating from the epiblast. Next, the chorion and allantois fuse, allowing the invagination of blood vessels through the chorionic layer, a critical process in generating the basic structure of the labyrinth layer of the placenta. Further expansion of this placental layer along with the differentiation of trophoblasts occurs. Throughout gestation, a continuous communication between the maternal decidua and the trophoblast cells is critical for a normal developmental progression. The trophoblast cells are key players in the process of placentation due to their capacity to invade the maternal uterine tissue upon implantation and remodel the vasculature that will form the basis of the placental vascular bed allowing for blood flow and nutrient exchange between the mother and the fetus [68].

In mice, the placenta is fully formed at embryonic (E) day 14.5, and is comprised of three distinct functional layers, namely the decidua, the junctional zone, and the labyrinth zone (Figure 2) [69]. The decidua is a maternal tissue that becomes specialized through decidualization during pregnancy. This tissue layer provides structural support to anchor the placenta to the uterus and immune tolerance toward the semi‐allogeneic fetus. The junctional zone lies between the decidua and the labyrinth zone and constitutes the primary endocrine site of the placenta [70, 71]. The labyrinth zone lies beneath the junctional zone and is characterized by an intricate architecture of a complex network of trophoblasts and maternal blood sinusoids, and fetal blood capillaries, together maximizing interhaemal transfer, allowing the fetus to grow in a timely manner [72, 73].

Despite the relatively short lifespan of the placenta in relation to an individual's life, its proper development and function are critical to arbitrate developmental outcomes and long‐term health trajectories. Recent work highlights the previously under‐appreciated involvement of the placenta in contributing to the integrity of the embryo and the development of various organ systems: gene knockouts that are associated with embryonic lethality also elicit placental defects, and conditional knockout experiments have demonstrated that a large fraction of embryonic defects can be rescued when providing the mutant embryo with a functional placenta [74]. Indeed, this extraembryonic tissue produces a variety of hormones, neurotransmitters, and growth factors that are critical for the development of the brain, heart, and vascular organ systems [75, 76, 77]. In fact, gene deletions that affect placentation are disproportionately accompanied by heart, brain, and vasculature system defects compared to knockouts that do not cause placental defects. In addition to supporting the development of various organ systems, the placenta plays critical roles in controlling fetal growth trajectories, which are thought to regulate metabolic programming. In particular, placental insufficiency is associated with fetal growth restriction (FGR), a recognized risk factor for metabolic and neurodevelopmental disorders. Indeed, FGR causes small‐for‐gestational‐age fetuses and babies to be born small, which are more likely to undergo a phase of “catch‐up growth,” a phase characterized by an imbalanced accumulation of lean‐to‐fat mass ratios associated with insulin insensitivity [78, 79, 80, 81, 82, 83].

## 5 Paternal Factors and Implications on Placental and Pregnancy Outcomes

The Great Obstetrical Syndromes encompass a range of pregnancy complications, including preeclampsia, intra uterine growth restriction (IUGR), preterm birth, and miscarriage. These conditions affect 10%–20% of pregnancies and are largely attributed to abnormalities in placental development and function [84, 85, 86]. Such pregnancy complications can not only be detrimental for the mother and the fetus, but can also have long term consequences on fetal metabolic programming. In addition to well‐established maternal risk factors, growing evidence point towards the contribution of the paternal genome, sperm epigenome, and semen composition, on placental development. Early evidence arose from foundational studies using uniparental mouse embryos which demonstrated that both maternal and paternal genomes are required for the completion of embryogenesis and proper placental formation [87, 88]. Notably, these experiments revealed that embryos with only maternal genomes lack TE development, whereas embryos derived from only paternal genomes fail to form ICM structures, suggesting diverging developmental biases whereby the maternal genome preferentially supports ICM formation and the paternal genome promotes TE development [89, 90, 91]. The complementary roles of the maternal and paternal genomes during development are conferred via epigenetic modifications such as genomic imprinting, leading to parent‐of‐origin‐specific gene expression. This functional asymmetry is further supported by transcriptomic and DNA methylation profiling of trophoblast cells derived from reciprocal horse‐donkey hybrids, which revealed that paternal alleles more strongly influence placental gene expression [92]. Additionally, recent work showed that histone methylation retained in mouse sperm correlate with TE and placenta epigenetic and transcriptomic profiles [17]. These studies provide a potential mechanistic link between paternal epigenetic information in sperm and early placental programming.

An increasing number of animal studies and human data suggest that paternal factors influence pregnancy outcomes via placental functions. In rodent models, paternal preconception exposure to toxicants or folate deficiency, as well as advanced paternal age or dysbiosis, have been linked to differential transcriptomic and epigenetic profiles in the placenta as well as offspring phenotypes [93, 94, 95, 96]. Additionally, paternal heat exposure as well as low‐protein diets have been linked to differential placental zone area sizes [25, 97]. In paternal preconception diet‐induced obesity models, paternal obesity has been associated with a reduced ICM:TE cell allocation ratio in blastocysts, suggesting an impairment in cell lineage specification [21, 98]. At mid‐ and late‐gestation stages, paternal diet‐induced obesity was linked to altered placental gene expression, with elevated hypoxia markers, impaired vasculature, and differential cellular composition [16, 18, 98]. Importantly, these paternally induced placental features and transcriptomic profiles corresponded to hypoxia‐induced placental insufficiency and growth restriction [18].

Collectively, these emerging pieces of evidence point toward a previously underappreciated paternal contribution to pregnancy outcomes, with the placenta as a potential site of action where environmentally‐induced changes on the sperm epigenome and also seminal fluid composition could act.

## 6 Candidate Genes of Diet‐Induced Sperm Epigenetics Regulate Placenta Phenotypes

Alterations in placental morphology, vascular organization, and overall size are central indicators of impaired placental function, often linked to disrupted nutrient and oxygen exchange [99], a hallmark of pregnancy complications such as fetal growth restriction and preeclampsia [100, 101]. Notably, defects in chorioallantoic fusion, a critical early event in placental formation, suggest profound developmental disruptions and are often embryonically lethal [102]. More subtle phenotypes, such as a pale placenta, reflect compromised perfusion or oxygenation [103, 104], while broader changes in placental physiology can encompass hormone production, immune modulation, and transport capacity, which may impact various organ system development, underscoring the organ's multifaceted role in sustaining pregnancy, growth, and development [99, 100, 105].

**FIGURE 3:**
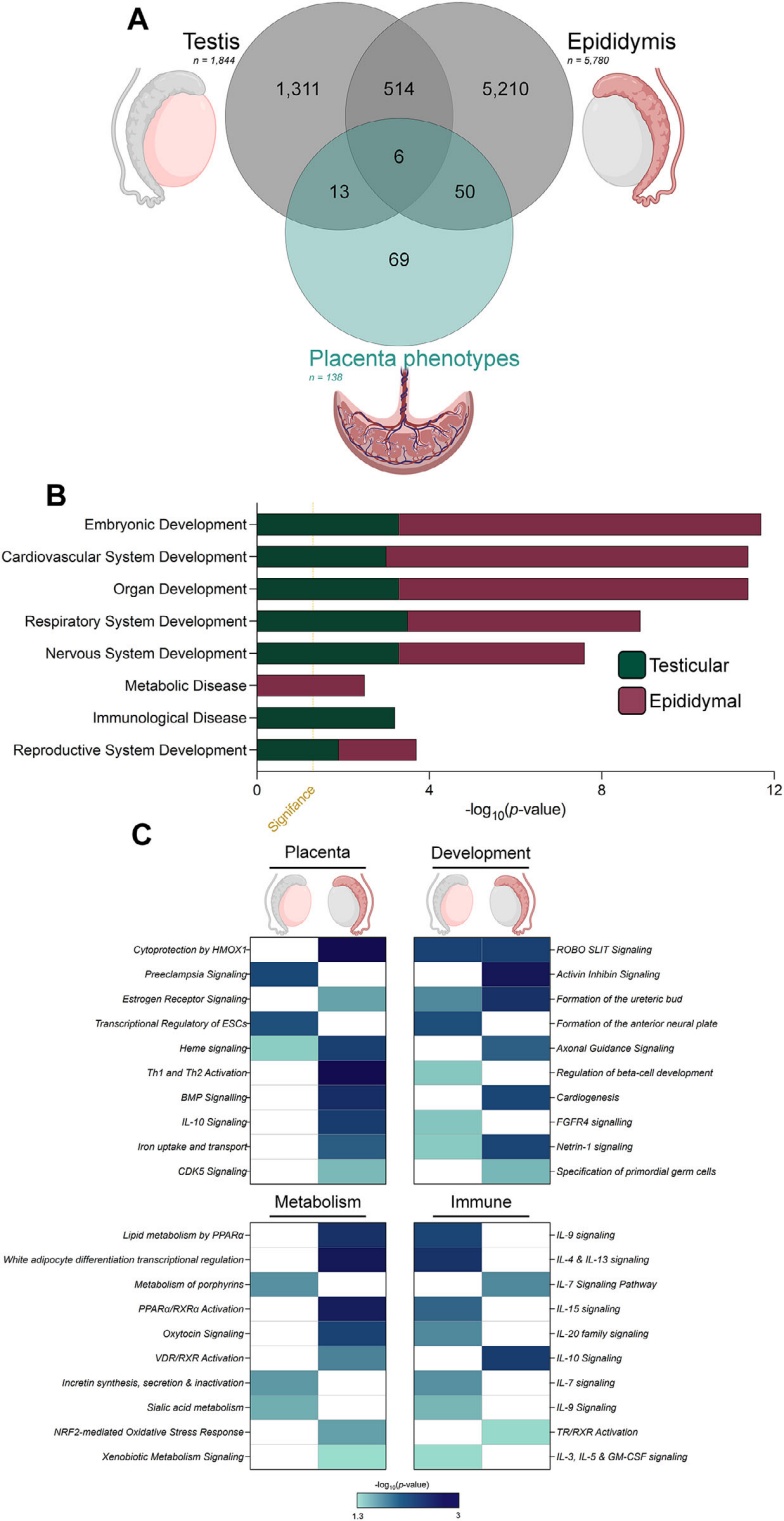
Sperm epigenome regulators of the placenta. (A) Venn Diagram overlap of placenta phenotype significant genes (*p* value ≤ 0.0001) with HFD altered genes via the testis and epididymis. (B) Top 8 diseases and functions categories identified by Ingenuity Pathway Analysis (*p ≤* 0.05) and (C) four dominant categories of canonical pathway.

To further highlight potential connections between diet‐induced changes in the sperm epigenome and placental functions that could impact offspring metabolic phenotypes, we integrated findings from key sperm HFD studies [17, 18, 19, 31, 32, 33, 34] with those of The International Mouse Phenotyping Consortium (IMPC). From these sperm studies, we compiled 1844 and 5780 high‐confidence genes from those that were testis‐ [17, 18] or epididymis‐derived [19, 31, 32, 33, 34], respectively (Figure 3A, Tables S1 and S2) (see Methods section for data inclusion, handling, and stringency). From the IMPC, we compiled 138 genes that exhibit placental phenotypes, some genes with multiple phenotypes (Table S3). Investigating the overlap of these sperm epigenome datasets with placenta phenotype genes, we uncovered 19 genes shared with testis‐derived changes and 56 genes from epididymal miRNA targets associated with the same placenta phenotypes (Figure 3A). Among the IMPC phenotypes linked exclusively to epididymal‐derived sperm signals, the sole hit was *placenta development*, driven by the knockout of cytochrome c oxidase subunit 7C (*Cox7c*), a mitochondrial electron‐transport gene essential for placental bioenergetic homeostasis [106]. To further expand on these candidate regulators, we employed ingenuity pathway analysis (IPA) (Testis, 19 genes; Epididymis, 56 genes). A comparative analysis revealed significant enrichment (*p* value ≤ 0.05) for developmental regulation across multiple systems, including cardiovascular, embryonic, nervous, metabolic, and reproductive systems (Figure 3B). Interestingly, epididymal contributions harbored greater significance across all categories, but exhibited a shared level for metabolic disease and slightly less for immunological disease (Table S4). Notably, *morphogenesis* and *size of embryo* functions were uniquely enriched in the epididymal contributions (1.84 × 10^−8^ and 1.01 × 10^−2^, respectively), supporting studies which suggest a epididymal miRNAs may play a role in early developmental programming [20, 24, 25].

To explore regulatory networks, we conducted pathway enrichment analyses, revealing four dominant biological themes; placenta, development, metabolism and immunology (Figure 3C, Table S5). In support of the regulation of placenta, we observed significant enrichment of pathways important in placenta development and vasculature, heme oxygenase 1 (HMOX1) [107, 108] and heme signaling [109]. Intriguingly, only testicular contributions were linked to preeclampsia, a pregnancy complication responsible for >570 000 deaths across mothers, fetus and neonates annually [110, 111, 112]. These findings, whilst speculative, raise the intriguing possibility that paternal preconception health may contribute to otherwise unexplained cases of placental dysfunction and preeclampsia [113]. Beyond the placenta, there are several pathways linked to development, a recurring highlight was nervous system regulation, in particular *ROBO/SLIT signaling*, an important regulator of neural tube development [114, 115], and *formation of the anterior neural plate* (Figure 3C). These associations suggest that paternal influences could potentially modulate neurodevelopmental trajectories, perhaps mediated through the placenta's regulation of brain development [75, 77], though this hypothesis will require further empirical validation. Interestingly, *regulation of beta‐cell development* was a significantly enriched function, supporting a possible mechanistic link between paternal diet to glucose intolerance in the next generation [19]. In concordance with this notion, amongst the metabolism pathways were *white adipocyte differentiation transcriptional regulation* and *PPARα/RXRα Activation*, important pathways in maintaining systemic insulin sensitivity, adipose tissue function, and hepatic lipid metabolism [116, 117, 118]. Lastly, in the immune landscape, we identified prominent enrichment for *interleukin signaling* (IL‐4, ‐7, ‐9, ‐13, ‐15, ‐20), hinting at an immunomodulatory axis of paternal influence on the fetal environment, a critical feature of implantation and placentation [119].

The interactive digital application, ShinySpermPlacenta introduced here, will help facilitate open‐access exploration of the paternal epigenetic data discussed here. This ShinyApp allows researchers to extract all HFD sperm epigenetic data and explore the overlap with the placenta phenotype‐driven genes (Figure 3A). Further, users will be able to explore the in‐silico outputs related to these overlapping genes (Figure 3B,C), filter for genes of interest with heatmaps responding in live time. Together, ShinySpermPlacenta will promote systems‐level understanding of how preconception environments shape the next generation.

These findings underscore the critical need to consider paternal nutritional status as a determinant of placental health and offspring metabolic outcomes. Historically, maternal contributions have dominated the research focus on the developmental origins of health and disease, nearly twentyfold more than paternal health [2, 14]. However, this data‐driven review underscores the sperm epigenome as an influential and biologically relevant contributor to early developmental programming. These findings add to a growing body of evidence suggesting that paternal health [120], too, shapes offspring outcomes in meaningful ways, offering a promising avenue for both mechanistic insights and preventative strategies.

## 7 Conclusion and Future Perspectives

The hypothesis that paternal pre‐conceptional health has a substantial intergenerational impact has been accumulating supporting evidence over the last two decades [32, 55, 56, 121, 122, 123]. Nonetheless, the current literature remains limited on interpretation, causality, reproducibility, and mechanistic depth. Further research on these aspects is of utmost importance to generate translational data that can support clinical applications and thereby help re‐balance pre‐conceptional health priorities from largely mono‐ to—at least—bi‐parental.

The evidence presented in this review contributes to the shift in how we conceptualize the role of paternal health in shaping offspring development and their metabolic trajectory. Far from being a passive carrier of DNA, sperm is increasingly recognized as an epigenetically active player, capable of transmitting nuanced signals about the father's nutrition and metabolic state to the next generation. These signals appear to influence not only early embryonic development but also the trajectory of placental formation, and long‐term metabolic health [19, 20, 25].

We want to highlight that paternal HFD exposure can reprogram the sperm epigenome, altering the expression of genes and sncRNAs that are implicated in placental development and metabolic disease susceptibility in offspring. These molecular changes, observed across both the testis and epididymis, impact pathways associated with vascular development, morphogenesis, immune modulation, and glucose regulation. This reveals a multi‐layered mechanism through which paternal diet influences intrauterine conditions and placental functions, independent of maternal contributions, and directly shapes the metabolic landscape of the developing fetus.

This growing body of evidence presents an opportunity to reframe preconception care as a truly bi‐parental responsibility. Although maternal nutrition remains central to healthy pregnancies, there is now strong justification for including paternal nutritional assessments and interventions in clinical practice [124]. This is best exemplified by the growing relationship between obesity and poor sperm parameters, including sperm concentration and motility,[125, 126, 127], which can be improve through diet and lifestyle changes [128], although this is of course multifactorial (reviewed in detail here [129, 130]). Incorporating fathers into the preconception health conversation could not only improve fertility outcomes but also reduce the burden of pregnancy complications and chronic disease in offspring [131]. The sperm epigenome itself holds promise as a biomarker rich platform for assessing reproductive risk and guiding personalized interventions.

Although not integrated in this article, it is important to consider that non‐germ cell factors, such as components present in the seminal fluid, may also influence the transmission of paternally‐induced traits. Experimental ablation of seminal vesicles has been associated with reduced fertility, compromised embryo development, placental overgrowth, and long‐term metabolic alterations in offspring [132]. These seminal fluid‐associated outcomes are thought to result from effects on sperm viability as well as through the modulation of the maternal reproductive tract environment [26, 132, 133, 134]. Evidence from preclinical studies indicates that obesity alters seminal fluid composition, which in turn can impact sperm quality and function [132, 135, 136, 137]. Nonetheless, the influence of seminal fluid alone is likely insufficient to account for the transmission of metabolic phenotypes, as in vitro fertilization (IVF)‐derived offspring generated from gametes of high‐fat‐fed sires display comparable metabolic impairments to those of offspring derived from natural mating [138]. Disentangling the relative contributions of sperm‐borne epigenetic information and seminal fluid‐derived signals remains an important avenue for future research, particularly in the context of paternal obesity, where combined effects may amplify risks to offspring health [17]. Beyond these seminal fluid‐mediated influences, sperm continue to be functionally and molecularly remodeled after ejaculation through extracellular vesicle (EV) interactions within the female reproductive tract. Proteomic and functional work has shown that supplementing bovine oviductosomes improves IVF rates and early embryo quality [139], while recent human data reveal region‐specific EVs from endometrial stromal cells, cervicovaginal and follicular fluid, that selectively boost progressive motility and acrosome reaction [140]. Complementary EVs in uterine fluid (uterosomes) have been shown to significantly enhance the rate of acrosome reaction in ejaculated human sperm [141]. Bidirectional exchange is also evident at the gamete interface, where CD9‐rich oocyte‐membrane fragments are actively incorporated into the sperm head prior to fusion, a trogocytosis‐like event thought to prime the sperm membrane for successful fertilization [142]. Although a comprehensive synthesis of these female‐tract EV dynamics lies outside the scope of this review, their inclusion here underscores an emerging, post‐ejaculatory layer of soma‐to‐germline communication that parallels the epididymal pathway described for the male. Furthermore, the epigenetically sensitive windows extend beyond the immediate preconception period. In males, neonatal and pre‐/peripubertal phases, when the germline undergoes extensive epigenetic reprogramming and expansion, appear particularly susceptible [143, 144, 145]. In females, oocyte growth and folliculogenesis similarly provide prolonged windows for environmental modulation [146, 147, 148]. Clarifying how exposures during these earlier stages intersect with preconceptional influences will be essential for a biologically grounded framework of intergenerational risk.

Looking forward, personalized nutrition strategies for prospective fathers could become a powerful tool in reproductive and developmental medicine [128]. These approaches may include tailoring dietary recommendations based on metabolic biomarkers, sperm epigenetic profiles, and lifestyle factors, thereby optimizing both fertility and the long‐term health of future children [149, 150]. In tandem, greater awareness of paternal health's contribution to fetal programming may prompt broader shifts in public health messaging and resource allocation.

To build on these insights, future studies should aim to functionally validate candidate epigenetic regulators using in vivo embryo and placental models, with a focus on disentangling the unique contributions of testis‐ versus epididymis‐derived modifications, as well as that of the molecular composition of the semen. As well as mechanistic studies also longitudinal human studies linking paternal diet and sperm health to placental function and outcomes in the children, including metabolic, immune, and neurodevelopmental trajectories, are urgently needed. In addition, multi‐omic approaches integrating transcriptomics, methylomics, proteomics, and chromatin accessibility will be critical to map the full extent of paternal influence. Complementing these bulk omic approaches, emerging single‐cell omic techniques are beginning to facilitate molecular profiling at the resolution of individual spermatozoa, making it possible to quantify cell‐to‐cell heterogeneity in RNA and chromatin features that bulk methods obscure. Proof‐of‐principle datasets exist [151], but current techniques are suboptimal for the fragmented, largely non‐polyadenylated sperm transcriptome, underscoring the need for sperm‐tailored single‐cell protocols before routine, quantitative estimates of within ejaculate frequencies can be achieved. Further, interactions between diet, environmental exposures, and epigenetic machinery also warrant deeper exploration, especially in the context of global trends of obesity and metabolic disorders.

These findings reinforce the notion that sperm is not merely a vessel for genetic material, but a dynamic communicator of environmental history and health status. As such, paternal nutrition emerges as a key target in the effort to optimize not only reproductive success but also the lifelong health of children. Integrating paternal health into public health policy, fertility care, and early‐life interventions represents both a challenge and a profound opportunity for improving intergenerational health outcomes.

## Conflicts of Interest

The authors declare no conflicts of interest.

## Supporting information




**Supporting File 1**: mnfr70261‐sup‐0001‐Table S1.xlsx.

**Supporting File 2**: mnfr70261‐sup‐0002‐Table S2.xlsx.

**Supporting File 3**: mnfr70261‐sup‐0003‐Table S3.xlsx.

**Supporting File 4**: mnfr70261‐sup‐0004‐Table S4.xlsx.

**Supporting File 5**: mnfr70261‐sup‐0005‐Table S5.xlsx.

**Supporting File 6**: mnfr70261‐sup‐0006‐Table S6.xlsx.

## Data Availability

The application is available at https://reproproteomics.shinyapps.io/ShinySpermPlacenta/, and the full source code can be accessed on GitHub: https://github.com/DavidSBEire/ShinySpermPlacenta.
